# Supplementary material for: Identification of photoperiod-induced specific miRNAs in the adrenal glands of Sunite sheep (Ovis aries)
Source: Front Vet Sci. 2022 Jul 22;9:888207. doi: 10.3389/fvets.2022.888207 (PMC9354845; doi:10.3389/fvets.2022.888207)
Supplement: Supplementary file 1 [file Data_Sheet_1.ZIP › Supplementary file/miRDeep2 file.zip!/miRDeep2 file/Novel_Structure/SPLP42A3/Novel_329.pdf]

Provisional ID	:	NC_019479.2_34104
Score total	:	1.8
Score for star read(s)	:	-1.3
Score for read counts	:	0
Score for mfe	:	1.5
Score for randfold	:	1.6
Score for cons. seed	:	
Total read count	:	814
Mature read count	:	814
Loop read count	:	0
Star read count	:	0

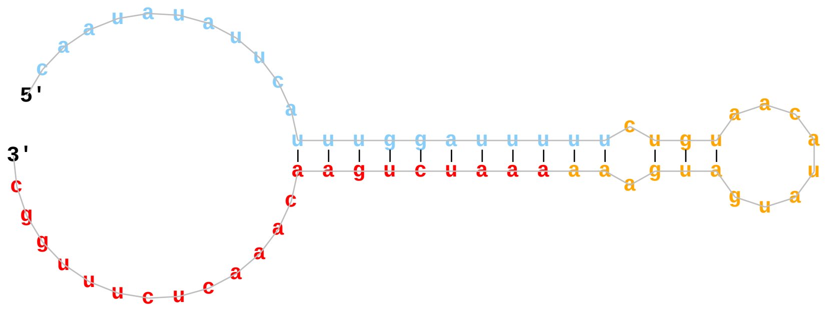

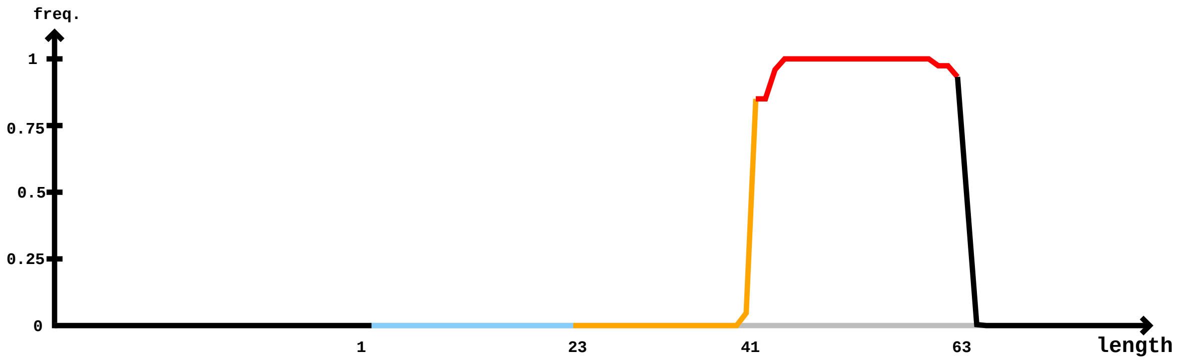

Star Mature

```
5'- ugcaagaucuggaacaagguauuggguuggccaauauauucauuuggauuuuucuguaacauaugaugaaaaaaucugaacaaacucuuuggccaacccaauacuaugagga   -3'    exp
    ..............((.(((((((((((((((((........(((((((((((.(((........))).)))))))))))........))))))))))))))))).))....    reads     mm        sample
    ......................................................................aaaaucugaacaaacuUuuu......................    18        1         seq
    ......................................................................aaaaucugaacaaacuUuuugg....................    19        1         seq
    ......................................................................aaaaucugaacaaacucuuugg....................    1         0         seq
    .......................................................................aaaucugaacaaacuUuuu......................    3         1         seq
    .......................................................................aaaucugaacaaacuUuuugg....................    14        1         seq
    .......................................................................aaaucugaacaaacuUuuuggc...................    637       1         seq
    .........................................................................aucugaacaaacuUuuuggc...................    16        1         seq
    .........................................................................aucugaacaaacuUuuuggcc..................    73        1         seq
    ..........................................................................ucugaacaaacuUuuuggcc..................    30        1         seq
    ..........................................................................ucugaacaaacuUuuuggcca.................    3         1         seq
```
